# Recent Advances in Green Synthesis, Characterization, and Applications of Bioactive Metallic Nanoparticles

**DOI:** 10.3390/ph15040455

**Published:** 2022-04-08

**Authors:** Shabaaz J. P. Begum, S. Pratibha, Janhvi M. Rawat, Divya Venugopal, Prashant Sahu, Abhilash Gowda, Kamal A. Qureshi, Mariusz Jaremko

**Affiliations:** 1 Department of Life Sciences, Graphic Era Deemed to be University, Dehradun 248002, India; shabaazbegumjp777@gmail.com (S.J.P.B.); janhvi.mishra03@gmail.com (J.M.R.); dvenugopal@yahoo.com (D.V.); 2 Department of Physics, BMS Institute of Technology and Management, Bengaluru 560064, India; 3 Babulal Tara Bhai Institute of Pharmaceutical Sciences, Sagar 470228, India; prashantsahu487@yahoo.com; 4 Bangalore Medical College and Research Institute, Bengaluru 560002, India; abhi1999blr@gmail.com; 5 Department of Pharmaceutics, Unaizah College of Pharmacy, Qassim University, Unaizah 51911, Saudi Arabia; ka.qurishe@qu.edu.sa; 6 Smart-Health Initiative (SHI) and Red Sea Research Center (RSRC), Division of Biological and Environmental Sciences and Engineering (BESE), King Abdullah University of Science and Technology (KAUST), Jeddah 23955, Saudi Arabia; mariusz.jaremko@kaust.edu.sa

**Keywords:** metallic nanoparticles, plant-mediated green synthesis, biofunctional, antimicrobial activity

## Abstract

Nanoparticles (NPs) are elements derived from a cluster of atoms with one or more dimensions in the nanometer scale in the range of 1–100 nm. The bio nanofabrication of metallic NPs is now an important dynamic area of research, with major significance in applied research. Biogenic synthesis of NPs is more desirable than physical and chemical synthesis due to its eco-friendliness, non-toxicity, lower energy consumption, and multifunctional nature. Plants outperform microorganisms as reducing agents as they contain large secondary biomolecules that accelerate the reduction and stability of the NPs. The produced NPs can then be studied spectroscopically (UV-Visible, XRD, Raman, IR, etc.) and microscopically (SEM, TEM, AFM, etc.). The biological reduction of a metallic ion or its oxide to a nanoparticle is quick, simple, and may be scaled up at room temperature and pressure. The rise in multi-drug resistant (MDR) microbes due to the immoderate use of antibiotics in non-infected patients is a major cause of morbidity and mortality in humans. The contemporary development of a new class of antibiotics with different mechanisms of action to kill microbes is crucial. Metals and their oxides are extremely toxic to microbes at unprecedentedly low concentrations. In addition, prevailing infections in plants and animals are raising significant concerns across the globe. NPs’ wide range of bioactivity makes them ideal antimicrobial agents in agricultural and medical fields. The present review outlines the synthesis of metallic NPs from botanicals, which enables the metals to be in a stabilized form even after ionization. It also presents a valuable database on the biofunctionalization of synthesized NPs for further drug development.

## 1. Introduction

The discovery of the fascinating properties of NPs has sparked an explosion of interest in nanotechnology. Nanotechnology is the manipulation of matter on a very small scale, typically between 1–100 nanometers [1,2,3,4]. For decades, it has been known that the effectiveness of biomaterials markedly depends upon their size. Applications corresponding to bulk materials have markedly decreased due to the present scenario corresponding to the plethora of nano applications. The main difference is that the fraction of atoms markedly increases in NPs than in microparticles or bulk elements. Due to their wholly new or enhanced capabilities based on size, distribution, and morphology, novel uses of NPs and nanomaterials are rapidly fast increasing on numerous fronts from the era onwards [5]. 

Phytonanotechnology has opened up new opportunities for the benign creation of NPs. Massive secondary biomolecules present in plants that are absent in microbes ensure that metals are reduced to their respective oxides, resulting in NPs of radically novel shapes and sizes [6,7,8,9,10]. The eco-friendly tasks in chemistry and its technologies are becoming distinctly recognizable and increasingly required due to unsustainable technologies available, which are concerned with environmental contamination concerns [11,12,13,14]. An increase in the population leads to a rapid increase in urbanization and industrialization, causing the dumping of more chemical waste and the deterioration of the earth due to the dumping of more chemical wastes. Exploration of nature and its natural products that follow the principle of green chemistry are more suitable for synthesizing NPs as they are sustainable, recyclable, free from contamination, and consume less energy. Biogenic synthesis has surpassed physical and chemical approaches in terms of reduced environmental effect and producing vast numbers of clean and sustainable NPs with well-defined size and form [15]. UV irradiation, aerosol technologies, laser ablation, ultrasonic fields, photochemical reduction, sol-gel process, heat evaporation, and electrochemical reduction techniques have successfully contributed to the production of NPs. However, they remain expensive and involve the use of toxic chemicals associated with the risks of precursor contamination, solvent toxicity, and hazardous byproduct formation [16,17,18,19,20] and, in most cases, to avoid nanoparticle aggregation. Organic passivators such as thiophenol, thiourea, mercapto acetate, etc., are woefully toxic to the environment, enough to cause pollution when produced in bulk. The greener route is the most propitious method for nanoparticle synthesis as it exploits the biological resources, including bacteria, fungi, viruses, algae, plants, and their extracts [21,22,23]. 

Since the 1970s, novel metal NPs have gained much attention due to their wide range of applications in medicine, biology, material science, physics, and chemistry. Due to their unique optical, electrical, chemical, photo-electrochemical, catalytic, magnetic, antibacterial, and biological labeling properties, the phytofabrication of metallic NPs, particularly noble metals, has exploded [24,25,26]. We now have a clear understanding of how size, shape, composition, crystallinity, and structure affect the inherent properties of novel metal NPs. Their remarkable characteristic distinguishes novel semiconductor metallic NPs from their bulk state. 

Bionanofabrication of NPs is a paradigm of a bottom-up approach that utilizes a biological system or its part as a reductant for the synthesis of NPs. The biogenic synthesis of metallic NPs using bacteria, fungi, plants, and biological compounds has been reported [27]. Plant diversity results from thousands of years of evolution; plants have evolved various mechanisms, including phytochemicals, to acclimate and survive across many domains and ecological situations. An enormous number of reports are staunched to the green synthesis of NPs with the various mechanisms of formation. Plant-mediated synthesis of biocompatible NPs is a one-step protocol and has advantages over microbe-mediated synthesis due to the presence of phytochemicals.

Additionally, using a prokaryotic organism is time-consuming due to the cultivation process, and there is a risk that microbes may lose viability and become mutated. The creation of NPs relies heavily on green chemistry. Green reactions and technologies that follow the 12 principles of green chemistry can assist in helping reduce pollution in the environment. This is performed by selecting elements such as capping agents/stabilizers, reducing agents, and solvents. An excellent alternative to the first two factors is the use of biogenic sources to synthesize NPs [28,29,30]. The overwhelming demand for reliable and eco-friendly methods can be fulfilled using bio-assisted photo resources. The phytochemicals such as alkaloids, amides, glycosides, flavonoids, polyphenols, quinones, saponins, tannins, and terpenoids cause the reduction of metals to their respective oxides. Organic agents obtained from plants and their parts act as reducing and stabilizing agents. Practically, bionanofabrication of NPs is efficiently conducted at room temperature and pressure. One-step synthesis of NPs is a simple process that involves adding extract and metallic salt, where the reduction and oxidation processes lead to the formation of nanostructures within minutes [31,32,33]. Despite a great deal of research in nanotechnology using physicochemical approaches, the synthesis of silver (Ag) and gold (Au) NPs is widely exploited using green synthesis. However, a relatively modest number of studies have attempted to elucidate the biosynthesis and potential applications of other metallic and semiconductor NPs [34,35,36,37,38]. 

The advancement of biologically based experimental procedures for the synthesis of NPs is seen as a critical milestone in nanotechnology. Considering the vast potentiality of plants as sources, this present review article has three objectives. First, to outline the exploration of plants and their extracts to design biofunctional metallic NPs using different metallic salts. The second part provides information about the various characterization tools used to identify synthesized NPs. Finally, the third part explains the biofunctionalization of the green synthesized NPs.

## 2. Synthesis of NPs

### 2.1. Perspectives of Nanoparticle Synthesis

**Figure 1:**
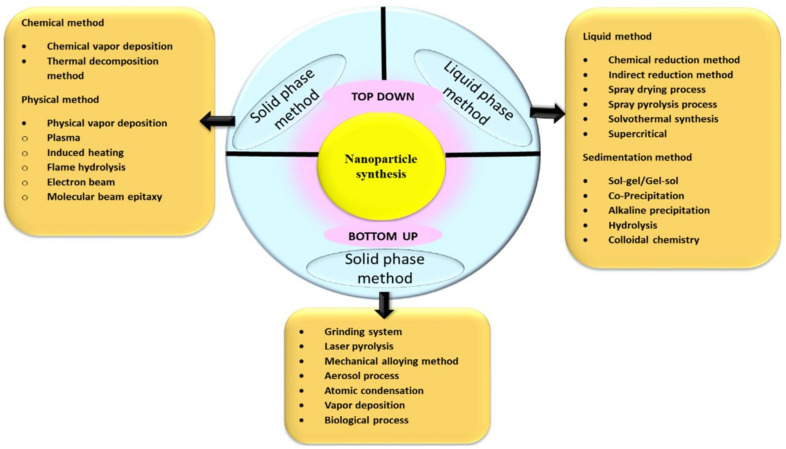
Various approaches for the synthesis of NPs.

The methodology for making ultrafine NPs from ancient times is generally by the breakdown (top-down), and the build-up (bottom-up) approaches, as illustrated in Figure 1. 

The breakdown approach of NP synthesis is usually employed during NPs’ physical and chemical synthesis. The size reduction of bulk material is used as a precursor ultimately to the nanosize by applying physical forces such as grinding, pulverization, etc., in the break down method which is also sometimes called the mechanochemical method [39]. It is challenging to obtain NPs by applying physical forces; usually, microparticles are easily obtained of 3 µm size, which is not significant. The second approach for obtaining NPs is by the build-up process; where major preparation methods for the synthesis of NPs can be achieved in two states of matter, liquid phase and solid phase, without any hazardous chemicals in biogenic synthesis, and remarkable increased use of chemicals in chemical synthesis are used. Biogenic synthesis of NPs falls under the bottom-up approach, where the uses of the biological system or its parts can be seen in the synthesis. To select the best organisms or extracts, one must evaluate their specific properties such as biochemical pathways, phytochemical contents, enzyme activities, cell growth circumstances, and ideal reaction [40].

### 2.2. Secondary Biomolecules for Capping and Stabilization

**Figure 2:**
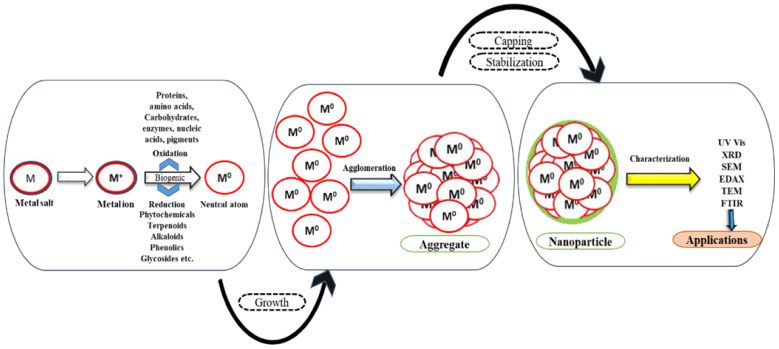
Biological reduction of NPs.

Plant extract not only acts as a reductant but also functions as a capping and stabilizing agent, as depicted in Figure 2.

Prediction of biomolecules acting as capping and stabilizing agents was realistic when IR spectrum of tea extract [41] showed the involvement of polyphenols, carboxylic acid, polysaccharide, amino acid, and proteins when coordinated with FTIR analysis. Zinc oxide NPs [ZnO NPs] showed peaks in 682–457 cm^−1^, indicating the presence of a higher percentage of phenolics. The stability studies of silver NPs (Ag NPs) synthesized from *Ziziphora tenuior* extract at room temperature revealed that bionanofabrication of Ag NPs was due to some metabolite functional groups such as amines, alcohols, ketones, aldehydes, and carboxylic acid. A peak graph of FTIR between the treated and untreated sample showed significant changes and predicted amide group form of proteins possibly be the covering layer of metal NPs [42]. The FTIR peak stretches in the OH, CH, C=C ring, and CH_2_ wagging of ascorbic acid indicated that *Hibiscus cannabis* extract comprises ascorbic acid responsible for reducing Ag NPs [43]. The ferric chloride test of coconut shell extract revealed the presence of phenolic compounds; most importantly, benzoquinone yielded the formation of Au NPs. *Calotropis gigantea*, a large shrub, consists of phytoconstituents such as cardiac glycosides, β-sitosterol, saponins, alkaloids, tannins, trisaccharides, and flavanols FTIR spectra denote the interactions of the biomolecules with Ag NPs [44].

## 3. Structural Analysis of NPs

The synergistic synthesized metallic NPs are characterized by assessment of their shape, size, morphology, and surface area, using various characterization tools such as Ultraviolet-visible spectrophotometry (UV-Vis), X-ray diffraction (XRD), Energy dispersive X-ray analysis (EDAX), Particle size distribution (PDS), Zeta potential (ZP), Photoluminescence (PL), Dynamic light scattering (DLS), Raman spectroscopy (R), Infrared spectroscopy (IR), Cyclic voltammetry (CV), Nanoparticle tracking analysis (NTA), Fourier transform-infrared spectroscopy (FTIR), Thermal gravimetric analysis (TGA), Selected area electron diffraction (SAED), Atomic field microscopy (AFM), Scanning electron microscope (SEM), field emission scanning electron microscopy (FESEM), Transmission electron microscopy (TEM), High-resolution transmission electron microscopy (HRTEM) as mentioned in Table 1. 

Characterization of a particular biomaterial depends on the complexity of the matrix, the analyte concentration, and the physio-chemical composition [45].

The UV-visible spectroscopy is primarily used as a characterization technique soon after synthesizing NPs of size from 2–100 nm in the range wavelength of 300–800 nm. The brownish color change of grapefruit extract from yellow due to the formation of silver ion complex was confirmed when a broad surface plasmon resonance band was observed around 450–470 nm [35]. Magnesium oxide NPs (MgO NPs) cubic structures formed in the presence of reductant *Emblica officinalis* [46]. The peak intensity profile was characteristic of NPs calculated with Scherer’s formula. The crystalline size determined for the mean particle size of the MgO NPs was around 27 nm which was well matched with SEM images. Every element in its unblended form will have unique atomic structures with a set of peaks EDAX can identify. A spectrum of Ag NPs observed at ~3 Kev confirmed the presence of silver as a major constituent element [47]. The elemental composition of Ag NPs synthesized from the plant extract of *Boerhaavia diffusa* was resolute by SEM equipped with an EDAX detector showing a strong signal in the silver region [48]. The stability of Ag NPs was evaluated by a zeta potentiometer. It was noted that synthesized NPs were stable in a wide range of pH from 6–12. An increase in the pH increased ZP. At pH 12, Ag NPs were found to be more stable. Raman spectroscopy of Ag NPs was carried out to gain the information of bio components initially for the biosynthesis [49] such as the polyphenol interactions with S^+^ ions during nanoparticle formation. Diluted samples of Ag NPs subjected to NTA reveal minimum and maximum 28 and 22 nm with a standard deviation of ±8 nm, and the results correlated with TEM findings were consistent [50,51]. Studies of possible biomolecules responsible for capping and stabilization of NPs were carried out by FT-IR and GC-MS analysis. *Cocos nucifera* coir extract confirmed the presence of biomolecules containing hydrocarbon such as nonacosane and heptacosames, which were predicted in the stabilization of Ag NPs by GC-Ms analysis [52]. The thermal gravimetric analysis allowed the study of the thermal stability of palladium NPs (Pd NPs) and showed that the phytoconstituents are responsible for reducing Pd^+2^ to Pd^o^ [53]. AFM characterized at ambient temperature exemplified the results of particles of 41 nm [24]. SEM images of carbon stretches provide a morphologically excellent view of NPs. Only TEM can reveal the exact shape and size of physical, chemical, or bio reduced NPs. TEM pictures of Ag NPs showed that most particles were spherical and measured between 5 and 20 nm [54].

## 4. Biofunctionalization of NPs

### 4.1. Gold NPs (Au NPs)

As the years have passed, gold has become a scarce commodity. Gold, a soft yellow metal with the highest ductility and malleability of any metal, is highly prized for various reasons; however, its physical qualities are critical to modern society’s functioning. In the treatment of rheumatic diseases and discoid lupus erythematosus, restorative dentistry, and other inflammatory skin conditions such as pemphigus urticaria and psoriasis, gold and its compounds were utilized [55]. Due to their small size, gold NPs (Au NPs) have a much higher surface area and dispersion. In terms of textural qualities, gold has the highest specific surface of any metal. 

Plant-based Au NPs are biocompatible and have unique chemical and optical properties, making them useful for photo-thermal treatment, bio-sensing, antioxidants, anti-microbials, and drug delivery [56,57,58,59,60,61,62,63,64,65,66,67,68,69,70]. Plant-Au NPs with surface modifications have been used in biomedical research and treatment [71,72,73,74,75]. Plant-Au NPs can interact with bacteria’s biomolecules, altering their structure and causing them to die. Plant-Au NPs, for example, were synthesized using flower extract of *Musa acuminate Colla* as a stabilizer and reducing agent [60] and displayed anticancer activity in addition to antibacterial activity against beta-lactamase-producing bacteria. Endocytosis allowed the plant-Au NPs to penetrate the cells after accumulating on the cell surface. On the surface of the Au NPs, free radicals were produced. Electron spin resonance spectroscopy revealed the formation of free radicals on the surface of Au NPs. When Au NPs were cultured with bacteria, the generation of free radicals indicated the ability of the Au NPs to disrupt cell membranes and make cells permeable, eventually leading to cell death. Plant antioxidants play a critical role in their biological competence, including interfering in cancer formation (including instigation, development, advancement, invasion, and metastasis) [61]. Plant extract-based Au NPs use free radicals and ROS in live cells in an antioxidant application [62]. When evaluated with 2, 2-diphenyl-1-picrylhydrazyl, the *Thymus vulgaris* aqueous extract was used to produce Au NPs [63], which exhibits antioxidant activity. The therapeutic impact on diabetic and obese rats using Au NPs derived from *Smilax glabra* was examined [64], and *Smilax glabra* rhizome extract capped Au NPs were generated. Histopathological investigations demonstrated the antidiabetic and antiobesity properties of plant based-Au NPs which reinstated the nuclei, inner membrane, and cytoplasm. Furthermore, *Vetex negundo* extract-stabilized Au NPs [73] and Au NPs capped using leaves extract of *Camellia sinensis* [67] were employed to cure severe myeloid leukemia in animal samples throughout pro-apoptotic evaluation of human gastric cancer cells, respectively.

A low-cost approach of gold NPs (Au NPs) at room temperature using aqueous seed extract of *Abelmoschus esculentus* yielded spherical particles, a narrow size range of 45–75 nm with a high antifungal effect against *Puccinia graminis* and *Candida albicans* [40]. As the demand for synthesis for NPs increased, various parts of the plants were utilized. *Ananas comosus* blended fruit extracts served as an excellent reductant for synthesizing Au NPs, having better antimicrobial activity than standard antibiotics used [75]. Microwave-mediated synthesis of Au NPs with coconut water is an incredible example of rapid NP synthesis, with an optimum time of 17 s. Furthermore, cytotoxicity was tested on two human cancer cell lines, HeLa (human cervical cancer) and MCF-7 (human breast cancer), and found to be nontoxic [12]. Leaf extract of *Ficus benghalensis* used as a capping agent in synthesis yielding spherical shape of Au NPs; the TEM analysis showed the formation of well-dispersed Au NPs of size 17–50 nm [76]. Au NPs from *Ficus religiosa* extract showed excellent stability and uniform capping due to the presence of polyphenols, amines and carboxylates and were nontoxic to the HEK293 cell lines at 80 µM concentration [77]. Nanotriangles and nanohexagons Au NPs of ~10 nm obtained from *Gnidia glauca* flower extract exhibit chemocatalytic activity in reducing 4-nitrophenol to 4-aminophenol by NaBH_4_ in the aqueous phase. In vitro antibacterial properties of polyshaped Au NPs synthesized from *Senna siamea* showed significant antibacterial activity against *Pseudomonas aeruginosa* (*P. aeruginosa*) [78]. Detailed view of functionalization of Au NPs mentioned in Table 2.

### 4.2. Silver NPs (Ag NPs)

In the current situation, numerous publications have been published on the laboratory-scale synthesis of Ag NPs from plants, which have emerged as antibacterial agents due to their unique physical and chemical properties. “Nobel silver NPs” are working to push the boundaries of science and technology, notably in the medical field [5]. Ag NPs have attracted intensive research in the biomedical, food industry, drug delivery, agriculture, water treatment, textile industries, antimicrobial agent, and anticancer drugs. A detailed view of functionalization of Ag NPs is mentioned in Table 3. 

Anti-angiogenic properties of Ag NPs in the rat aortic ring model were evaluated. Results showed that Ag NPs at 200 µg/mL led to a 50% reduction in the length and number of vessel-like structures [13]. *Artocarpus heterophyllus* assisted Ag NPs reduction, showing excellent antibacterial activity against *Staphylococcus aureus* (*S. aureus*) with an inhibition zone of 15 mm diameter compared to the other bacterial strains used [37]. Antibacterial activity of phytosynthesized Ag NPs from *Boerhaavia diffusa* L. extract were tested against fish bacterial pathogens *Aeromonas hydrophila*, *Flavobacterium branchiophilum*, and *Pseudomonas fluorescens*; and they demonstrated high antibacterial activity towards *Flavobacterium branchiophilum* when compared to other two fish bacterial pathogens [48]. FTIR research revealed that protein fractions acted as reducing and stabilizing agents during the production of Ag NPs using latex from *Calotropis gigantea* L. [44]. In situ green synthesis of Ag NPs was done using *Coriandrum sativum* L. seed extract, then exposed to synergistic tests. The results showed that the potency of conventional antibiotics could be increased in the presence of Ag NPs. Green Ag NPs reduced using *Cocos nucifera* coir extract were effective anti-larvicidal agents against *C. quinquefasciatus* and *Anopheles stephensi* [52]. Ag NPs serve as an excellent substitute besides synthetic and chemical insecticides synthesized from *Euphorbia hirta* L. extract, and administration of it to the crop pest cotton bollworm *Helicoverpa armigera* impacted its biological parameters such as less consumption of food index due to a decrease in the level of digestive enzymes. Systematic evaluation of antibacterial properties of Ag NPs synthesized from extracts of *Hibiscus cannabis* [98], *Moringa oleifera* L. [21], *Prosporis fracta* [91], *Pterocarpus santalinus* [30], and *Vitis vinifera* against common human pathogens such as *Klebsiella pneumoniae (K. pneumoniae*), *Escherichia coli* (*E. coli*), *Enterococcus faecalis*, *Enterobacter cloacae* (*E. cloacae*), *Proteus vulgaris*, *S. aureus*, *S. saprophyticus*, *Bacillus subtilis*, and *P. aeruginosa* gave a brief insight on eradicating the conventional antibiotics and exploring more therapeutic applications of Ag NPs as nanoantibiotics against MDR bacteria [29]. Extensive study of bioactivity of Ag NPs against breast cancer cell lines MCF 7 interestingly displayed a decrease in the cell viability. Direct sunlight assisted Ag NPs within five minutes, providing better fungicidal activity against *Candida albicans*, *Candida glabrata*, and *Aspergillus niger*. Ag NPs’ biological activity from *Illicium verum* Hook. [54], was not reported.

### 4.3. Platinum Group of Metals

Platinum is a silvery-white metal costlier than gold, known for its malleability, durability and ductility. The first paper on Pt NPs using leaf extract described the use of an aqueous leaf solution of *Diospyros kaki* as a bio-reducing agent in the green extracellular synthesis of Pt NPs from an aqueous H_2_PtCl_6_.6H_2_O solution [99]. With a reaction temperature of 95 °C, platinum ions’ conversion to NPs greater than 90% was accomplished. The mechanism of action was unknown at the time, but the FTIR study indicated that Pt NPs are surrounded by metabolites such as terpenoids, which have functional groups of amines, alcohols, ketones, aldehydes, and carboxylic acids. A single-step technique was proposed for synthesizing Pt NPs using an invasive weed, *Lantana camara*, and a moderate reducing agent, ascorbic acid [100]. The procedure entails mixing adequate amounts of platinum (VI) solution, *Lantana* leaf extract, and ascorbic acid, then heating the mixture to 95 °C for 8 min. Ascorbic acid and leaf extract combined with decreasing chloroplatinic acid to platinum NPs, with leaf extract also serving as a stabilizing agent for the NPs. The resultant NPs are 35 nm in diameter and crystallize in face-centered cubic symmetry. Palladium is a sister metal of platinum and belongs to the platinum group metals (PGM). It has a white luster finish and is light. However, it is less ductile than platinum. 

Palladium NPs (Pd NPs), which are important due to their catalytic characteristics and affinity for hydrogen, have been phyto-sensitized using *Solanum trilobatum* under contemporary pH and room temperature which exhibited antibacterial and cytotoxic effects [101]. Pd NPs have also been used as a homogeneous or heterogeneous catalysts in various scientific fields, including hydrogen storage, chemo-optical transducers, and chemical modifiers in ETV-ICP-MS, hydrogen sensor, automotive catalytic converter, plasmonic waveguides, and optical limiting devices. For the production of Pd NPs using diverse concentrations and temperatures, an eco-friendly and cost-effective green technique using water-soluble leaf extract of *Sapium sebiferum* as a reducing and capping agent was developed. The optimized Pd NPs synthesized using 10 mL leaf extract showed strong bacterial inhibition against *P. aeruginosa* (11 ± 0.6 mm), *Bacillus subtilis* (19 ± 0.6 mm), and *S. aureus* (29 ± 0.8 mm) [102]. Uniform-sized palladium NPs were synthesized using *Curcuma longa* tuber extract with an average size ranging between 10–15 nm [103]. Liquid flower extracts of *Moringa oleifera* flower extracts with 1 mM palladium acetate solution yielded 10–50 nm size bio Pd NPs [104]. GC-MS analysis showed that Bis-phthalate was mostly the reducing agent. Pd NPs caused significant cytotoxicity to A549 cells and did not induce toxicity in normal healthy peripheral lymphocytes. Table 4 depicts the bio-reduction of platinum group NPs and their bioactivity.

### 4.4. Metallic Oxide NPs

Different types of metal oxides synthesized using the plant parts are useful for bioactivity, which are discussed in the succeeding sections in detail. The bio-reduction of other metal oxides NPs and their bioactivity is listed in Table 5.

#### 4.4.1. Zinc Oxide NPs (ZnO NPs)

Zinc oxide (ZnO) is an inorganic yellow amorphous solid powder. Highly ionic zinc oxide NPs are one-of-a-kind in that they can be made with a large surface area and a strange crystal structure and size [105]. On an important note, ZnO NPs are advantageous nanoantibiotics compared to other metal and metal oxide NPs due to their cost effectiveness, white appearance, antimicrobial, and UV blocking properties. Some studies indicated high specific toxicity of ZnO NPs against bacteria, and only minimal effects were observed on human cells [3]. Green synthesis of ZnO NPs using aqueous leaves extract *Camellia sinensis* recorded the formation of hexagonal wurtzite bionanomaterials of 16 nm-sized ZnO NPs exhibited excellent antimicrobial activity against bacteria and fungi compared to the synthetic antibiotic [106]. Fatty acid in the *Anchusa italica* flower extract yielded ~8–14 nm in size. ZnO NPs had antibacterial efficacy against both Gram-positive and Gram-negative bacteria, and cytotoxicity experiments on Vero cells revealed dose-dependent toxicity with no harm at concentrations < 142 µg/mL [107]. Nanoscale antimicrobial ZnO NPs formed using bark *Boswellia ovalifoliata* as reductant showed enhanced antimicrobial activity against isolated bacterial and fungal scales formed in drinking water PVC pipelines [108]. 

#### 4.4.2. Magnesium Oxide NPs (MgO NPs)

Magnesium oxide (MgO) is a fascinating basic metal oxide with many uses. MgO, for example, has shown significant potential as a destructive adsorbent for harmful chemical agents due to its ultrafine, nanoscale particles and high specific surface area. Because of its particular architectures, nanoscale MgO has unique optical, electrical, magnetic, thermal, mechanical, and chemical capabilities [109]. The 27 nm of crystalline size of MgO NPs is obtained from the reduction of magnesium nitrate hexahydrate in the presence of ethanolic fruit extract of *Emblica officinalis* [110]. The antibacterial activity MgO NPs treated on cotton fabric recommended that these particles can be used in the preparation of fabric used in surgical clothes, wound dressing and bandages of bed lining activity. *Clitoria ternatea* acted as a stabilizer for MgO NPs, which exhibited good antioxidant activity [42].

#### 4.4.3. Copper Oxide NPs (CuO NPs)

Copper oxide (CuO) nanostructures offer a wide range of applications, including high-Tc superconductors, sensors, catalytic, optical, electrical, gigantic magnet resistance materials, gas sensors, solar energy conversion, and the preparation of organic-inorganic nanostructured composite. CuO has a bandgap of 1.7 eV and is a p-type semiconductor. It can also be used as an antibacterial agent. A green chemistry approach to reducing CuO NPs using aquatic noxious weed *Eichhornia crassipes* gave rise to stable spherical NPs of 28 ± 4 mm size [111]. Antifungal activity of CuO NPs against selected plant pathogenic fungi showed the highest inhibition zone of 21.26 ± 1 mm diameter for *Fusarium culmorum* compared to the standard antibiotic used having inhibition zone of 19.33 ± 1 mm. CuO NPs generated by solution combustion utilizing *Gloriosa superba* extract as fuel had a considerable antibacterial impact on selected bacterial strains such as *Klebsiella aerogenes*, *E. coli*, *Staphylococcus aureus*, and *Pseudomonas desmolyticum* [112].

#### 4.4.4. Titanium Dioxide NPs (TiO_2_ NPs)

Titanium dioxide (TiO_2_) has become part of our everyday lives. It is found in various consumer goods and products of daily use such as cosmetics, paints, dyes and varnishes, textiles, paper and plastics, food and drugs, and even paving stones. A total of 4.68 million tons of titanium dioxide was produced worldwide in 2009. Nanoscale TiO_2_ NPs are manufactured by using plant source extract of *Psidium guajava*. Phenolic content present in the leaves reduced metatitanic acid to titanium dioxide. Determination of antibacterial activity against test organisms created a great conflict of interest when it showed more inhibition zone than the standard antibiotic tetracycline [113]. Treatment failure is frequently caused by insecticide resistance and a failure to follow the application directions for topical pediculicides. A study was conducted to determine the larvicidal and pediculicidal activity of synthesized TiO_2_ NPs against the fourth instar larvae of the malaria vector, *Anopheles subpictus grassi*, the head louse, *Pediculus humanus capitis* (De Geer) (Phthiraptera: Pediculidae) as well as against the filariasis vector, *Culex quinquefasciatus* Say (Diptera: Culicidae). The researchers discovered that the TiO_2_ NPs they created have outstanding mosquito larvicidal and anti-lice properties [114].

#### 4.4.5. Lanthanide NPs

A typical member of the lanthanide series can withstand significantly high temperatures above 700 °C. Lanthanide radionuclides are well-known in nuclear medicine because they can detect and cure malignant tumors. The ability to generate controlled-size lanthanide NPs opens up a wide range of applications in nuclear medicine because the dosages are directly related to the number of unstable atoms involved. 

Samarium nitrate along with *Medicago sativa* (alfa-alfa) was subjected for the synthesis of NPs at pH 4, 6, 7 and 8. Unagglomeratized 10 nm size stable samarium NPs were obtained at pH 4; in contrast, at pH 6, 7 and 8, samarium NPs were found to be agglomerated and of irregular sizes [115]. 

Neodymium, a soft silvery metal used in the commercial preparation of glass dyes and its alloys, is used to prepare magnets used in microphones, professional loudspeakers, and ear headphones. Nanostructures of neodymium were prepared using the bioreduction method. Alfa-alfa leaf extract was used to reduce Nd^3+^ ions to Nd^0^ [116]. The perspectives of application in electronic, photonic, and even nuclear medicine can be associated with the properties of these lanthanide elements and also to the zero- and one-dimension electron confinement that nanostructures reveal. The bio-reduction of all the above-discussed metal oxide NPs and their bioactivity is given in Table 5.

**Table 5:** Bio-reduction of other metal oxides NPs and their bioactivity.

Sr. No.	Botanical Names of Plants	Part Used	Size Range (nm) (SEM/TEM)	Characterization Tools	Bio-Functionalization	Ref.
Zinc oxide NPs						
**1.**	*Trianthema* *portulacastrum*	Extract	25–90	UV–Vis, XRD, FTIR, SEM, TEM, XPS	Cytotoxic Cytotoxic Antibacterial Antifungal Antioxidant	[117]
**2.**	*Matricaria chamomilla* L., *Lycopersicon* *esculentum M.*, *Olea* *europaea*	Extract	40.5–124	UV–Vis, XRD, FTIR, SEM, TEM, EDS	Antibacterial	[118]
**3.**	*Punica granatum*	Extract	32.98–81.84	UV–Vis, XRD, FTIR, SEM, TEM	Cytotoxic Antibacterial	[119]
**4.**	*Rheum turketanicum*	Extract	17–20	UV–Vis, XRD, FTIR, SEM, TEM	Cytotoxic	[120]
**5.**	*Tecoma castanifolia*	Extract	70–75	UV–Vis, XRD, FTIR, SEM, TEM	Antibacterial Antioxidant Anticancer	[121]
**6.**	*Silybum marianum*	Extract	31.2	UV–Vis, XRD, FTIR, SEM, TEM	Antifungal Antibacterial Cytotoxic	[122]
**7.**	*Anchusa italic*	Flower	~8–~14	UV-Vis, EDX XRD, FT-IR, FESEM, TEM	Antibacterial Cytotoxic	[107]
**8.**	*Aloe vera*	Leaves	8–20	UV-Vis, EDX, XRD, FT-IR, GC-MS, SEM TEM	Antibacterial Cytotoxic	[3]
**9.**	*Rosa canina*	Fruit	50–400	XRD, EDX, DLS, FT-IR, SEM	Antibacterial Antioxidant Cytotoxic	[36]
**10.**	*Boswellia ovalifoliata*	Bark	20	UV-Vis, DLS, ZP, FTIR, SEM, TEM	Antimicrobial	[108]
Magnesium oxide NPs						
**1.**	*Emblica officinalis*	Fruit	27	UV-Vis, XRD, EDX, FT-IR, SEM	Antibacterial	[110]
**2.**	*Clitoria ternatea*	Whole plant	50–400 nm	UV-Vis, XRD, PL, FTIR, EDS, FESEM	Antioxidant	[42]
Copper oxide NPs						
**1.**	*Ocimum tenuiflorum*	Extract	20–30 nm	UV–Vis, XRD, FTIR, SEM, TEM	Antibacterial	[123]
**2.**	*Moringa oleifera*	Extract	35–95 nm	UV–Vis, XRD, FTIR, SEM, TEM	Antifungal	[124]
**3.**	*Eichhornia crassipes*	Leaves	28 ± 4	UV-Vis, XRD, FT-IR, FESEM	Antifungal	[111]
**4.**	*Gloriosa superba*	Leaves	5–10	UV-Vis, PXRD, SEM TEM	Antibacterial	[112]
Titanium dioxide NPs						
**1.**	*Artocarpus heterophyllus*	Extract	15–20 nm	UV–Vis, XRD, FTIR, SEM, TEM	Anticancer Cytotoxic Antibacterial	[125]
**2.**	*Citrus sinensis*	Fruit peel	20–50 nm	UV–Vis, XRD, FTIR, SEM, EDAX, TEM	Anticancer Cytotoxic Antibacterial	[126]
**3.**	*Musa alinsanaya*	Fruit peel	31.5 nm	UV–Vis, XRD, FTIR, SEM, EDAX, TEM	Larvicidal antibacterial	[114]
**4.**	*Psidium guajava*	Leaves	32.58	XRD, EDX, FT-IR, FESEM	Antibacterial Antioxidant	[113]
**5.**	*Vitex negundo*	Leaves	93.33	UV-Vis, XRD, EDX, FTIR, SEM	Antibacterial	[4]
Samarium NPs						
**1.**	*Medicago sativa*	leaves	10	UV-Vis	Antitumor	[115]
Neodymium NPs						
**1.**	*Medicago sativa*	Leaves	10	UV-Vis, RS, PSD, DLS, EDAX, XRD, FT-IR, SEM	NR	[116]

Note: NR = Not reported.

## 5. Applications of Phytofabricated NPs

### 5.1. In Agriculture

Antimicrobials based on NPs have an impact because of their extensive physiochemical properties in terms of size, shape, surface area, surface energy, crystallinity, charge, aggregation, agglomeration, and chemical composition.

In previous investigations, the microbicidal activities of several inorganic NPs such as TiO_2_, Ag, CuO, MgO, S, and ZnO were examined separately or in combination with biopolymer [127,128,129]. As a result, it is important to create the new green synthesis-based NPs capable of managing fungal phytopathogens through biofunctionalized antimicrobial NPs to protect plants in a cost-effective, environmentally friendly, and long-term manner.

### 5.2. Applications of Phytofabricated NPs as Nanoantibiotics

Over past decades, resistance to antibiotics has become increasingly widespread and resulted in noteworthy deaths of humans. The emergence and re-emergence of pathogens have become a major public health concern worldwide, and the rapid emergence of antibiotic-resistant Gram-positive and Gram-negative pathogenic germs is a major public health concern [130,131,132,133,134]. The long list of drug-resistant bacteria includes macrolide-resistant *Streptococcus pyogenes*, sulfonamide, penicillin, methicillin-resistant *Staphylococcus aureus* (*MRSA*), vancomycin-resistant *Enterococcus*, penicillin-resistant *Streptococcus pneumoniae*, multi-drug resistant *Mycobacterium tuberculosis* (*MDR-M. tuberculosis*), penicillin-resistant *Neisseria gonorrhoeae (PRNG)*, *E. coli*, *E. cloacae*, *K. pneumoniae*, *Salmonella enterica*, *Shigella flexneri*, *Acinetobacter baumannii*, *Vibrio cholerae*, *P. aeruginosa*, and beta-lactamase-expressing *Haemophilus influenzae*.

## 6. Mode of Action of NPs

**Figure 3:**
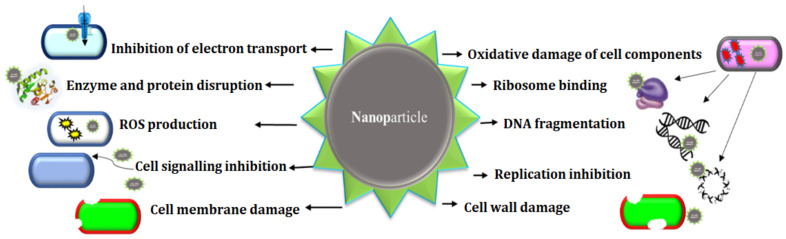
Schematic diagram of the mechanism of action of NPs against microorganisms.

Numerous studies demonstrate that metallic NPs harm microorganisms by forming reactive oxygen species (ROS) such as hydroxyl and superoxide radicals, causing DNA damage, oxidative damage, and cell wall and membrane damage [108]. The mechanism of action of NPs is schematically represented in Figure 3.

## 7. Discussion

In hospitals in the United States and the United Kingdom, 40–60 percent of *S. aureus* strains are resistant to methicillin (MRSA), and the majority of these infections are also resistant to several medicines. Drug resistance in bacteria has several severe consequences in medicine and society. Drug-resistant bacterial infections necessitate greater drug doses, the inclusion of more toxic therapies, more extended hospital stays, and a higher fatality rate. More deaths are linked to MRSA than methicillin-sensitive *S. aureus* (MSSA). Infections caused by antibiotic-resistant bacteria cost the US $20 billion in overall healthcare costs, plus an additional $35 billion in societal losses. Longer treatment times, higher healthcare costs, a high death rate, and a short life expectancy have resulted from this. In both community and hospital settings, more medicines to control and halt the spread of such illnesses are desperately needed. 

Another study concludes that increasing the percentage of plant extract in nanoparticle preparation reduces their antibacterial activities. NPs produced from antimicrobial chemicals may be made in a simple, straightforward, and cost-effective manner and are suitable for defining new categories of nanobiotic substances that might be employed as novel, environmentally friendly nano-antimicrobial agents. On the other hand, the size of metallic NPs affects their microbicidal activities; thus, concentration-dependent studies of metallic NPs biosynthesized under various reaction conditions can have significant technological implications, particularly in biomedical applications. Antimicrobial properties rise as metallic concentration increases, while bacterial and fungal growth speed decreases, which is consistent with prior research on nanoparticle influence on microorganisms. We can conclude with certainty that microbial harm may occur more quickly in the presence of NPs. This environmentally safe technique of synthesis and application of metallic NPs as microbicidal agents makes them promising candidates for biofilm removal in food packaging, waste-water treatment, and, most critically, the development of medicines against multidrug-resistant pathogens [135]. 

## 8. Conclusions and Future Perspectives

This review discusses the recent advances in green synthesis, characterization, and applications of bioactive metallic NPs. Additionally, it explains how plant-derived secondary metabolites such as alkaloids, terpenoids, quinones, and others are used as reducing and capping agents in these green approaches. These secondary metabolites are naturally produced from harmless plant materials such as plant extracts from numerous commonly accessible plants like bananas, tea, onion, and coconut. Green synthesis can overcome the avoidance of toxic or harsh chemicals; even production on a reasonable scale can be achieved. The primary advantage of biosynthesized NPs over physical and chemical ones is strict size control, improving their stability and longevity. Several methods have been implicated for easier nanoparticle synthesis based on the metal types. Bio functionalization of NPs is remarkable in in vitro studies, but there are lesser reports on in vivo studies due to its lesser-known mechanism of action. Biostatic analysis of exploited metals shows the enormous use of silver due to its rapid synthesis in the liquid phase and its synergistic effect against microbes. Nevertheless, the area-specific, varietal and seasonal variations in chemical constituents of plant extracts may often lead to different results in different laboratories, which warrant further studies in identifying key plant biomolecules or metabolites for developing more efficient technology for NP synthesis. Nanotechnology can increase food quality, global food production, plant protection, detection of plant and animal diseases, monitoring of plant growth, and reduce waste for “sustainable amplification”.

## Figures and Tables

**Table 1:** An overview of techniques to characterize physicochemical properties of NPs.

Techniques	Instruments	Mass Number	Number	Size Distribution	Agglomeration State	Shape	Surface Area	Chemical Composition
Spectroscopy techniques	UV visible Spectroscopy			✓				✓
	X-ray Diffraction			✓				✓
	FT-IR spectroscopy							✓
	RAMAN spectroscopy							✓
	Atomic absorption/optical emission spectroscopy							✓
	Mass spectroscopy	✓						✓
	X-ray photoelectron							✓
	Dynamic light Scattering			✓	✓			
	Zeta potential						✓	
Microscopy techniques	Scanning electron Microscopy		✓	✓	✓	✓		
	Transmission electron microscopy		✓	✓	✓	✓		
	Scanning probe microscopy		✓	✓	✓	✓		

**Table 2:** Bio-reduction of gold NPs and their bioactivity.

Sr.No.	Botanical Names of Plants	Part Used	Size Range (nm) (SEM/TEM)	Characterization Tools	Bio-Functionalization	Ref.
**1.**	*Ricinus communis*	Leaf	40–80	UV-Vis, XRD, FT-IR, TEM, HRTEM	Antimicrobial Anticancer	[79]
**2.**	*Anacardium occidentale*	Bark	10–60	XRD, FT-IR, UV-Vis, TEM	Antibacterial Anticancer	[80]
**3.**	*Dracocephalum kotschyi*	Flower	5–21	XRD, UV-Vis, FT-IR, TEM	Anticancer	[81]
**4.**	*Euphorbia peplus*	Fruit	50	XRD, FT-IR, TEM, UV-Vis	Anticancer Antibacterial Insecticidal	[82]
**5.**	*Gnidia glauca* L.	Flower	5–20	XRD, SEM, FT-IR, UV-Vis, TEM	Chemocatalytic	[83]
**6.**	*Abelmoschus* *esculentus* (L.) Moench	Seed	45–75	UV-Vis, EDX XRD, FT-IR, AFM, FESEM	Antifungal	[40]
**7.**	*Ananas comosus* (L.)	Fruit	10 ± 5	UV-Vis, EDX, XRD, FT-IR, SEM	Antimicrobial	[16]
**8.**	*Cocos nucifera* Linn.	Liquid endosperm	0.22	UV-Vis, XRD, FT-IR, AFM, TEM, HRTEM	Cytotoxicity	[12]
**9.**	*Ficus religiosa* L.	Bark	20–30	UV-Vis, XRD, FT-IR, TEM	MTT assay	[77]
**10.**	*Morinda citrifolia* L.	Root	12.17–38.26	UV-Vis, XRD, EDX, FT-IR, FE-SEM, TEM	NR	[84]
**11.**	*Pistia stratiotes* L.	Aerial and submerged parts	2–40	UV-Vis, XRD, EDAX, FT-IR, SEM, HR-SEM, TEM	NR	[7]
**12.**	*Senna siamea* (Lam.)	Leaf	70	UV-Vis, XRD, FT-IR, HRTEM	Antibacterial	[78]
**13.**	*Plumeria alba* Linn.	Flower	20–30	UV-Vis, HRTEM	Antimicrobial	[85]
**14.**	*Euphorbia fischeriana*	Root		UV-Vis EDX XRD, FT-IR, FESEM	Antioxidant	[71]

Note: NR = Not reported.

**Table 3:** Bio-reduction of silver NPs and their bioactivity.

Sr.No.	Botanical Names of Plants	Part Used	Size Range (nm) (SEM/TEM)	Characterization Tools	Bio-Functionalization	Ref.
**1.**	*Tropaeolum majus L*	Flower	38–82	UV–Vis, XRD, FTIR, SEM, TEM	Antibacterial	[86]
**2.**	*Camellia Sinensis*	Leaf	4–50	UV-Vis, XRD, FT-IR, TEM	Antibacterial	[87]
**3.**	*Ferula gumosa*, *Ferula* *latisecta*, *Teucrium* *polium*, *Trachomitum* *venetum*	Leaf	20–80	UV-Vis, XRD, EDX, AFM, FT-IR, SEM	Antibacterial	[88]
**4.**	*Juniperus procera*	Fruit	30–90	UV-Vis, XRD, EDX, FT-IR, SEM	Antibacterial	[89]
**5.**	*Holoptelea integrifolia*	Unripe fruits	32–38	UV-Vis, XRD, EDX, FT-IR, SEM, TEM	Anti-diabetic Antibacterial Anti-inflammatory	[90]
**6.**	*Prosopis farcta*	Leaf	11–15	UV-Vis, XRD, FT-IR, SEM, TEM	Antioxidant antibacterial	[91]
**7.**	*Piper longum* L.	Leaf	28.8	UV-Vis, XRD, EDX, FT-IR, TEM	Antioxidant Anti-cancer Anti-larvicidal	[92]
**8.**	*Solidago canadensis*	Latex	100–300	UV-Vis, XRD, EDAX, FT-IR, FESEM, TEM	Cytotoxic	[93]
**9.**	*Cocos nucifera* Linn.	Coir	23 ± 2	UV-Vis, XRD, GC-MS, TEM	Larvicidal	[52]
**10.**	*Coriandrum sativum* L.	Seed	13.09	UV-Vis, XRD, PDS, SEM, TEM	Antimicrobial	[94]
**11.**	*Euphorbia hirta* L.	Leaf	30–60	UV-Vis, EDS, FT-IR, SEM	Pesticidal	[24]
**12.**	*Musa acuminata colla* L.	flower	Nanoclusters	UV-Vis, XRD, FT-IR, EDS, SEM	Antibacterial Antifungal	[60]
**13.**	*Nelumbo nucifera* Gaertn.	Root	~16.7	UV-Vis, XRD, FT-IR, SEM, TEM	Protein binding Antioxidant Anticancer	[95]
**14.**	*Ocimum sanctum* Linn.	Leaf	50	UV-Vis, XRD, SEM	Antimicrobial	[96]
**15.**	*Withania somnifera* Linn.	Leaf	5–30	UV-Vis, IR, EDS, CV, NTA, ZP, FT-IR, TEM	Antimicrobial	[97]

**Table 4:** Bio-reduction of platinum group NPs and their bioactivity.

Sr. No.	Botanical Names of Plants	Part Used	Size Range (nm) (SEM/TEM)	Characterization Tools	Bio-Functionalization	Ref.
Platinum NPs						
**1.**	*Diospyros kaki*	Leaves	2–12	UV-Vis, XRS, FT-IR, HRTEM	NR	[99]
**2.**	*Lantana camara* (L.)	Leaves	35	UV-Vis, RS, PSD, DLS, EDAX, XRD, FT-IR, SEM	NR	[100]
Palladium NPs						
**1.**	*Sapium sebiferum*	Leaves	2–12	UV-Vis	Antibacterial	[102]
**2.**	*Moringa oleifera*	Leaves	35	UV-Vis, RS, PSD, DLS, EDAX, XRD, FT-IR, SEM	Antibacterial Antioxidant Anticancer	[104]

Note: NR = Not reported.

## Data Availability

Data sharing not applicable.

## References

[1] Ahmad S., Munir S., Zeb N., Ullah A., Khan B., Ali J., Ali S. (2019). Green nanotechnology: A review on green synthesis of silver nanoparticles—an ecofriendly approach. Int. J. Nanomed..

[2] Ahmed M.J., Murtaza G., Mehmood A., Bhatti T.M. (2015). Green synthesis of silver nanoparticles using leaves extract of *Skimmia laureola*: Characterization and antibacterial activity. Mater. Lett..

[3] Ali K., Dwivedi S., Azam A., Saquib Q., Al-Said M.S., Al-Khedhairy A., Musarrat J. (2016). Aloe vera extract functionalized zinc oxide nanoparticles as nanoantibiotics against multi-drug resistant clinical bacterial isolates. J. Colloid Interface Sci..

[4] Ambika S., Sundrarajan M. (2016). [EMIM] BF4 ionic liquid-mediated synthesis of TiO_2_ nanoparticles using *Vitex negundo* Linn extract and its antibacterial activity. J. Mol. Liquids.

[5] Amini S.M. (2019). Preparation of antimicrobial metallic nanoparticles with bioactive compounds. Mater. Sci. Eng. C.

[6] Anand K., Tiloke C., Phulukdaree A., Ranjan B., Chuturgoon A., Singh S., Gengan R. (2016). Biosynthesis of palladium nanoparticles by using *Moringa oleifera* flower extract and their catalytic and biological properties. J. Photochem. Photobiol. B Biol..

[7] Anuradha J., Abbasi T., Abbasi S.A. (2014). An eco-friendly method of synthesizing gold nanoparticles using an otherwise worthless weed pistia (*Pistia stratiotes* L.). J. Adv. Res..

[8] Arruda S.C.C., Silva A.L.D., Galazzi R., Azevedo R.A., Arruda M.A.Z. (2015). Nanoparticles applied to plant science: A review. Talanta.

[9] Ascencio J.A., Canizal G., Medina-Flores A., Bejar L., Tavera L., Matamoros H., Liu H.B. (2006). Neodymium Nanoparticles: Biosynthesis and Structural Analysis. J. Nanosci. Nanotechnol..

[10] Ascencio J.A., Rincon A.C., Canizal G. (2005). Synthesis and Theoretical Analysis of Samarium Nanoparticles: Perspectives in Nuclear Medicine. J. Phys. Chem. B.

[11] Azizi S., Ahmad M.B., Namvar F., Mohamad R. (2014). Green biosynthesis and characterization of zinc oxide nanoparticles using brown marine macroalga *Sargassum muticum* aqueous extract. Mater. Lett..

[12] Babu P.J., Das R.K., Kumar A., Bora U. (2011). Microwave-Mediated Synthesis of Gold Nanoparticles Using Coconut Water. Int. J. Green Nanotechnol..

[13] Baharara J., Namvar F., Ramezani T., Hosseini N., Mohamad R. (2014). Green Synthesis of Silver Nanoparticles using *Achillea biebersteinii* Flower Extract and Its Anti-Angiogenic Properties in the Rat Aortic Ring Model. Molecules.

[14] Baker S., Rakshith D., Kavitha K.S., Santosh P., Kavitha H.U., Rao Y., Satish S. (2013). Plants: Emerging as Nanofactories towards Facile Route in Synthesis of Nanoparticles. BioImpacts.

[15] Bar H., Bhui D.K., Sahoo G.P., Sarkar P., Pyne S., Misra A. (2009). Green synthesis of silver nanoparticles using seed extract of *Jatropha curcas*. Colloids Surf. A Physicochem. Eng. Asp..

[16] Basavegowda N., Sobczak-Kupiec A., Malina D., Yathirajan H.S., Keerthi V.R., Dinkar S. (2013). Plant mediated synthesis of gold nanoparticles using fruit extracts of *Ananas comosus* (L.) (pineapple) and evaluation of biological activities. Adv. Mater. Lett..

[17] Bayda S., Adeel M., Tuccinardi T., Cordani M., Rizzolio F. (2020). The History of Nanoscience and Nanotechnology: From Chemical–Physical Applications to Nanomedicine. Molecules.

[18] Das J., Das M.P., Velusamy P. (2013). Sesbania grandiflora leaf extract mediated green synthesis of antibacterial silver nanoparticles against selected human pathogens. Spectrochim. Acta Part A Mol. Biomol. Spectrosc..

[19] Chandra H., Kumari P., Bontempi E., Yadav S. (2020). Medicinal plants: Treasure trove for green synthesis of metallic nanoparticles and their biomedical applications. Biocatal. Agric. Biotechnol..

[20] Crisan C.M., Mocan T., Manolea M., Lasca L.I., Tăbăran F.-A., Mocan L. (2021). Review on Silver Nanoparticles as a Novel Class of Antibacterial Solutions. Appl. Sci..

[21] Das S., Parida U.K., Bindhani B.K. (2013). Green biosynthesis of silver nanoparticles using *Moringa oleifera* L. leaf. Int. J. Nanotechnol. Appl..

[22] De Oliveira P.F.M., Torresi R.M., Emmerling F., Camargo P.H.C. (2020). Challenges and opportunities in the bottom-up mechanochemical synthesis of noble metal nanoparticles. J. Mater. Chem. A.

[23] Deb S. (2014). Synthesis of silver nano particles using *Murraya koenigii* (Green Curry Leaves), *Zea mays* (baby corn) and its antimicrobial activity against pathogens. Int. J. PharmTech Res..

[24] Devi G.D., Murugan K., Selvam C.P. (2014). Green synthesis of silver nanoparticles using *Euphorbia hirta* (Euphorbiaceae) leaf extract against crop pest of cotton bollworm, *Helicoverpa armigera* (Lepidoptera: Noctuidae). J. Biopestic..

[25] Dikshit P., Kumar J., Das A., Sadhu S., Sharma S., Singh S., Gupta P., Kim B. (2021). Green Synthesis of Metallic Nanoparticles: Applications and Limitations. Catalysts.

[26] Dipankar C., Murugan S. (2012). The green synthesis, characterization and evaluation of the biological activities of silver nanoparticles synthesized from *Iresine herbstii* leaf aqueous extracts. Colloids Surf. B Biointerfaces.

[27] El-Seedi H.R., El-Shabasy R.M., Khalifa S.A.M., Saeed A., Shah A., Shah R., Iftikhar F.J., Abdel-Daim M.M., Omri A., Hajrahand N.H. (2019). Metal nanoparticles fabricated by green chemistry using natural extracts: Biosynthesis, mechanisms, and applications. RSC Adv..

[28] Chopade B.A., Ghosh S., Patil S., Ahire M., Kitture R., Jabgunde A., Kale S., Pardesi K., Cameotra S.S., Bellare J. (2012). Synthesis of silver nanoparticles using *Dioscorea bulbifera* tuber extract and evaluation of its synergistic potential in combination with antimicrobial agents. Int. J. Nanomed..

[29] Gnanajobitha G., Paulkumar K., Vanaja M., RajeshKumar S., Malarkodi C., Annadurai G., Kannan C. (2013). Fruit-mediated synthesis of silver nanoparticles using Vitis vinifera and evaluation of their antimicrobial efficacy. J. Nanostruct. Chem..

[30] Gopinath K., Gowri S., Arumugam A. (2013). Phytosynthesis of silver nanoparticles using *Pterocarpus santalinus* leaf extract and their antibacterial properties. J. Nanostruct. Chem..

[31] Gour A., Jain N.K. (2019). Advances in green synthesis of nanoparticles. Artif. Cells Nanomed. Biotechnol..

[32] Hassanisaadi M., Bonjar G., Rahdar A., Pandey S., Hosseinipour A., Abdolshahi R. (2021). Environmentally Safe Biosynthesis of Gold Nanoparticles Using Plant Water Extracts. Nanomaterials.

[33] Hussain I., Singh N.B., Singh A., Singh H., Singh S.C. (2016). Green synthesis of nanoparticles and its potential application. Biotechnol. Lett..

[34] Ibrahim H.M. (2015). Green synthesis and characterization of silver nanoparticles using banana peel extract and their antimicrobial activity against representative microorganisms. J. Radiat. Res. Appl. Sci..

[35] Jadoun S., Arif R., Jangid N.K., Meena R.K. (2020). Green synthesis of nanoparticles using plant extracts: A review. Environ. Chem. Lett..

[36] Jafarirad S., Mehrabi M., Divband B., Kosari-Nasab M. (2016). Biofabrication of zinc oxide nanoparticles using fruit extract of *Rosa canina* and their toxic potential against bacteria: A mechanistic approach. Mater. Sci. Eng. C.

[37] Jagtap U.B., Bapat V.A. (2013). Green synthesis of silver nanoparticles using *Artocarpus heterophyllus* Lam. seed extract and its antibacterial activity. Ind. Crop. Prod..

[38] Jahan I. (2022). Phyto-Nanofabrication: Plant-Mediated Synthesis of Metal and Metal Oxide Nanoparticles. Handbook of Research on Green Synthesis and Applications of Nanomaterials.

[39] Jamkhande P.G., Ghule N.W., Bamer A.H., Kalaskar M.G. (2019). Metal nanoparticles synthesis: An overview on methods of preparation, advantages and disadvantages, and applications. J. Drug Deliv. Sci. Technol..

[40] Jayaseelan C., Ramkumar R., Rahuman A.A., Perumal P. (2013). Green synthesis of gold nanoparticles using seed aqueous extract of *Abelmoschus esculentus* and its antifungal activity. Ind. Crops Prod..

[41] Jeevanandam J., Kiew S.F., Boakye-Ansah S., Lau S.Y., Barhoum A., Danquah M.K., Rodrigues J. (2022). Green approaches for the synthesis of metal and metal oxide nanoparticles using microbial and plant extracts. Nanoscale.

[42] Sushma N.J., Prathyusha D., Swathi G., Madhavi T., Raju B.D.P., Mallikarjuna K., Kim H.-S. (2015). Facile approach to synthesize magnesium oxide nanoparticles by using *Clitoria ternatea*—characterization and in vitro antioxidant studies. Appl. Nanosci..

[43] Jyoti K., Pattnaik P., Singh T. (2021). Green Synthesis of Silver Nanoparticles Using Sustainable Resources and their Use as Antibacterial Agents: A Review. Curr. Mater. Sci. Former. Recent Pat. Mater. Sci..

[44] Mathew S., Victorio C.P., Sidhi J., Thanzeela B.H.B. (2020). Biosynthesis of silver nanoparticle using flowers of *Calotropis gigantea* (L.) WT Aiton and activity against pathogenic bacteria. Arab. J. Chem..

[45] Kalimuthu K., Cha B.S., Kim S., Park K.S. (2019). Eco-friendly synthesis and biomedical applications of gold nanoparticles: A review. Microchem. J..

[46] Suresh J., Yuvakkumar R., Sundrarajan M., Hong S.I. (2014). Green synthesis of magnesium oxide nanoparticles. Advanced Materials Research.

[47] Karaiskos I., Giamarellou H. (2014). Multidrug-resistant and extensively drug-resistant Gram-negative pathogens: Current and emerging therapeutic approaches. Expert Opin. Pharmacother..

[48] Komal R., Arya V. (2013). Biosynthesis and characterization of silver nanoparticles from aqueous leaf extracts of *Carica papaya* and its antibacterial activity. Int. J. Nanomater. Biostruct..

[49] Kumara P.P.N.V., Pammib S.V.N., Kollu P., Satyanarayana K.V.V., Shameema U. (2014). Green synthesis and characterization of silver nanoparticles using *Boerhaavia diffusa* plant extract and their anti-bacterial activity. Ind. Crop. Prod..

[50] Lade B.D., Shanware A.S. (2020). Phytonanofabrication: Methodology and factors affecting biosynthesis of nanoparticles. Smart Nanosystems for Biomedicine, Optoelectronics and Catalysis.

[51] Farhadi S., Ajerloo B., Mohammadi A. (2017). Low-cost and eco-friendly phyto-synthesis of Silver nanoparticles by using grapes fruit extract and study of antibacterial and catalytic effects. Int. J. Nano Dimens..

[52] Roopan S.M., Madhumitha G., Rahuman A.A., Kamaraj C., Bharathi A., Surendra T.V. (2013). Low-cost and eco-friendly phyto-synthesis of silver nanoparticles using *Cocos nucifera* coir extract and its larvicidal activity. Ind. Crop. Prod..

[53] Fahmy S.A., Preis E., Bakowsky U., Azzazy H.M.E.-S. (2020). Palladium Nanoparticles Fabricated by Green Chemistry: Promising Chemotherapeutic, Antioxidant and Antimicrobial Agents. Materials.

[54] Luna C., Chávez V., Barriga-Castro E.D., Núñez N.O., Mendoza-Reséndez R. (2015). Biosynthesis of silver fine particles and particles decorated with nanoparticles using the extract of *Illicium verum* (star anise) seeds. Spectrochim. Acta Part A Mol. Biomol. Spectrosc..

[55] Qiao J., Qi L. (2020). Recent progress in plant-gold nanoparticles fabrication methods and bio-applications. Talanta.

[56] Zhang Y., Zhang C., Xu C., Wang X., Liu C., Waterhouse G., Wang Y., Yin H. (2019). Ultrasmall Au nanoclusters for biomedical and biosensing applications: A mini-review. Talanta.

[57] Xiao T., Huang J., Wang D., Meng T., Yang X. (2019). Au and Au-Based nanomaterials: Synthesis and recent progress in electrochemical sensor applications. Talanta.

[58] Shahriari M., Hemmati S., Zangeneh A., Zangeneh M.M. (2019). Biosynthesis of gold nanoparticles using *Allium noeanum* Reut. ex Regel leaves aqueous extract; characterization and analysis of their cytotoxicity, antioxidant, and antibacterial properties. Appl. Organomet. Chem..

[59] Gharehyakheh S., Ahmeda A., Haddadi A., Jamshidi M., Nowrozi M., Zangeneh M.M., Zangeneh A. (2020). Effect of gold nanoparticles synthesized using the aqueous extract of *Satureja hortensis* leaf on enhancing the shelf life and removing *Escherichia coli* O157:H7 and *Listeria monocytogenes* in minced camel’s meat: The role of nanotechnology in the food industry. Appl. Organomet. Chem..

[60] Valsalam S., Agastian P., Esmail G.A., Ghilan A.-K.M., Al-Dhabi N.A., Arasu M.V. (2019). Biosynthesis of silver and gold nanoparticles using *Musa acuminata colla* flower and its pharmaceutical activity against bacteria and anticancer efficacy. J. Photochem. Photobiol. B Biol..

[61] Zhaleh M., Zangeneh A., Goorani S., Seydi N., Zangeneh M.M., Tahvilian R., Pirabbasi E. (2019). In vitro and in vivo evalution of cytotocicity, antioxidant, antibacterial, antifungal, and cutaneous wound healing properies of gold nanoparticles produced via a green chemistry synthesis using *Gundelia tournefortii* L. as acapping and reducing agent. Appl. Organomet. Chem..

[62] Jeyarani S., Vinita N.M., Puja P., Senthamilselvi S., Devan U., Velangani A.J., Biruntha M., Pugazhendhi A., Kumar P. (2020). Biomimetic gold nanoparticles for its cytotoxicity and biocompatibility evidenced by fluorescence-based assays in cancer (MDA-MB-231) and non-cancerous (HEK-293) cells. J. Photochem. Photobiol. B Biol..

[63] Hemmati S., Joshani Z., Zangeneh A., Zangeneh M.M. (2019). Green synthesis and chemical characterization of *Thymus vulgaris* leaf aqueous extract conjugated gold nanoparticles for the treatment of acute myeloid leukemia in comparison to doxorubicin in a leukemic mouse model. Appl. Organomet. Chem..

[64] Ansari S., Bari A., Ullah R., Mathanmohun M., Veeraraghavan V.P., Sun Z. (2019). Gold nanoparticles synthesized with *Smilax glabra* rhizome modulates the anti-obesity parameters in high-fat diet and streptozotocin induced obese diabetes rat model. J. Photochem. Photobiol. B Biol..

[65] Ismail E.H., Saqer A.M.A., Assirey E., Naqvi A., Okasha R.M. (2018). Successful Green Synthesis of Gold Nanoparticles using a *Corchorus olitorius* Extract and Their Antiproliferative Effect in Cancer Cells. Int. J. Mol. Sci..

[66] Filip G.A., Moldovan B., Baldea I., Olteanu D., Suharoschi R., Decea N., Cismaru C.M., Gal E., Cenariu M., Clichici S. (2018). UV-light mediated green synthesis of silver and gold nanoparticles using Cornelian cherry fruit extract and their comparative effects in experimental inflammation. J. Photochem. Photobiol. B Biol..

[67] Ahmeda A., Zangeneh A., Zangeneh M.M. (2020). Green synthesis and chemical characterization of gold nanoparticle synthesized using *Camellia sinensis* leaf aqueous extract for the treatment of acute myeloid leukemia in comparison to daunorubicin in a leukemic mouse model. Appl. Organomet. Chem..

[68] Liu R., Pei Q., Shou T., Zhang W., Hu J., Li W. (2019). Apoptotic effect of green synthesized gold nanoparticles from *Curcuma wenyujin* extract against human renal cell carcinoma A498 cells. Int. J. Nanomed..

[69] Liu Y., Kim S., Kim Y.J., Perumalsamy H., Lee S., Hwang E., Yi T.H. (2019). Green synthesis of gold nanoparticles using *Euphrasia officinalis* leaf extract to inhibit lipopolysaccharide-induced inflammation through NF-kappa B and JAK/STAT pathways in RAW 264.7 macrophages. Int. J. Nanomed..

[70] Park S.Y., Yi E.H., Kim Y., Park G. (2019). Anti-neuroinflammatory effects of *Ephedra sinica Stapf* extract-capped gold nanoparticles in microglia. Int. J. Nanomed..

[71] Zhang T., Dang M., Zhang W., Lin X. (2019). Gold nanoparticles synthesized from *Euphorbia fischeriana* root by green route method alleviates the isoprenaline hydrochloride induced myocardial infarction in rats. J. Photochem. Photobiol. B Biol..

[72] Ahmeda A., Zangeneh M.M. (2019). Novel green synthesis of *Boswellia serrata* leaf aqueous extract conjugated gold nanoparticles with excellent anti-acute myeloid leukemia property in comparison to mitoxantrone in a leukemic mice model: Introducing a new chemotherapeutic drug. Appl. Organomet. Chem..

[73] Yun Z., Chinnathambi A., Alharbi S.A., Jin Z. (2019). Biosynthesis of gold nanoparticles using *Vetex negundo* and evaluation of pro-apoptotic effect on human gastric cancer cell lines. J. Photochem. Photobiol. B Biol..

[74] Siddiqi K.S., Husen A. (2017). Recent advances in plant-mediated engineered gold nanoparticles and their application in biological system. J. Trace Elements Med. Biol..

[75] Acharya D., Mohanta B., Pandey P. (2021). Green synthesis of Silver and Silver-gold core-shell nanoparticles using Pineapple leaf extract (*Ananas comosus*) and study of their antibacterial properties. Int. J. Nano Dimens..

[76] Francis G., Thombre R., Parekh F., Leksminarayan P. (2014). Bioinspired synthesis of gold nanoparticles using *Ficus benghalensis* (Indian Banyan) leaf extract. Chem. Sci. Trans..

[77] Wani K., Choudhari A., Chikate R., Kaul-Ghanekar R. (2013). Synthesis and characterization of gold nanoparticles using *Ficus religiosa* extract. Carbon Sci. Technol..

[78] Reddy G.R., Morais A.B., Gandhi N.N. (2013). Green Synthesis, Characterization and in vitro Antibacterial Studies of Gold Nanoparticles by Using *Senna siamea* Plant Seed Aqueous Extract at Ambient Conditions. Asian J. Chem..

[79] Ghramh H.A., Khan K.A., Ibrahim E.H., Setzer W.N. (2019). Synthesis of Gold Nanoparticles (AuNPs) Using *Ricinus communis* Leaf Ethanol Extract, Their Characterization, and Biological Applications. Nanomaterials.

[80] Sunderam V., Thiyagarajan D., Lawrence A.V., Mohammed S.S.S., Selvaraj A. (2018). In-vitro antimicrobial and anticancer properties of green synthesized gold nanoparticles using *Anacardium occidentale* leaves extract. Saudi J. Biol. Sci..

[81] Chahardoli A., Karimi N., Fattahi A., Salimikia I. (2018). Biological applications of phytosynthesized gold nanoparticles using leaf extract of *Dracocephalum kotschyi*. J. Biomed. Mater. Res. Part A.

[82] Ghramh H.A., Khan K.A., Ibrahim E.H. (2019). Biological Activities of *Euphorbia peplus* Leaves Ethanolic Extract and the Extract Fabricated Gold Nanoparticles (AuNPs). Molecules.

[83] Ghosh S., Patil S., Ahire M., Kitture R., Gurav D.D., Jabgunde A.M., Kale S., Pardesi K., Shinde V., Bellare J. (2012). *Gnidia glauca* flower extract mediated synthesis of gold nanoparticles and evaluation of its chemocatalytic potential. J. Nanobiotechnol..

[84] Suman T., Rajasree S.R., Ramkumar R., Rajthilak C., Perumal P. (2013). The Green synthesis of gold nanoparticles using an aqueous root extract of *Morinda citrifolia* L.. Spectrochim. Acta Part A Mol. Biomol. Spectrosc..

[85] Nagaraj B., Malakar B., Divya T.K., Krishnamurthy N.B., Liny P., Dinesh R. (2012). Environmental benign synthesis of gold nanoparticles from the flower extracts of *Plumeria alba* Linn, (Frangipani) and evaluation of their biological activities. Int. J. Drug Dev. Res..

[86] Valsalam S., Agastian P., Arasu M.V., Al-Dhabi N.A., Ghilan A.-K.M., Kaviyarasu K., Ravindran B., Chang S.W., Arokiyaraj S. (2018). Rapid biosynthesis and characterization of silver nanoparticles from the leaf extract of *Tropaeolum majus* L. and its enhanced in-vitro antibacterial, antifungal, antioxidant and anticancer properties. J. Photochem. Photobiol. B Biol..

[87] Göl F., Aygün A., Seyrankaya A., Gür T., Yenikaya C., Şen F. (2020). Green synthesis and characterization of *Camellia sinensis* mediated silver nanoparticles for antibacterial ceramic applications. Mater. Chem. Phys..

[88] Uttu A.J., Sallau M.S., Iyun O.R.A., Ibrahim H. (2022). Antimicrobial Efficacy of Selected *Strychnos* Species: A Mini Review. J. Chem. Rev..

[89] Ibrahim E., Kilany M., Ghramh H.A., Khan K., Islam S.U. (2018). Cellular proliferation/cytotoxicity and antimicrobial potentials of green synthesized silver nanoparticles (AgNPs) using *Juniperus procera*. Saudi J. Biol. Sci..

[90] Kumar V., Singh S., Srivastava B., Bhadouria R., Singh R. (2019). Green synthesis of silver nanoparticles using leaf extract of *Holoptelea integrifolia* and preliminary investigation of its antioxidant, anti-inflammatory, antidiabetic and antibacterial activities. J. Environ. Chem. Eng..

[91] Salari S., Bahabadi S.E., Samzadeh-Kermani A., Yosefzaei F. (2019). In-vitro Evaluation of Antioxidant and Antibacterial Potential of Green Synthesized Silver Nanoparticles Using *Prosopis farcta* Fruit Extract. Iran. J. Pharm. Res. IJPR.

[92] Yadav R., Saini H., Kumar D., Pasi S., Agrawal V. (2019). Bioengineering of *Piper longum* L. extract mediated silver nanoparticles and their potential biomedical applications. Mater. Sci. Eng. C.

[93] Botha T.L., Elemike E.E., Horn S., Onwudiwe D.C., Giesy J.P., Wepener V. (2019). Cytotoxicity of Ag, Au and Ag-Au bimetallic nanoparticles prepared using golden rod (*Solidago canadensis*) plant extract. Sci. Rep..

[94] Nazeruddin G., Prasad N., Shaikh Y., Waghmare S., Adhyapak P. (2014). *Coriandrum sativum* seed extract assisted in situ green synthesis of silver nanoparticle and its anti-microbial activity. Ind. Crop. Prod..

[95] Sreekanth T., Ravikumar S., Eom I.-Y. (2014). Green synthesized silver nanoparticles using *Nelumbo nucifera* root extract for efficient protein binding, antioxidant and cytotoxicity activities. J. Photochem. Photobiol. B Biol..

[96] Rout Y., Behera S., Ojha A.K., Nayak P.L. (2012). Green synthesis of silver nanoparticles using *Ocimum sanctum* (Tulashi) and study of their antibacterial and antifungal activities. J. Microbiol. Antimicrob..

[97] Raut R.W., Mendhulkar V.D., Kashid S.B. (2014). Photosensitized synthesis of silver nanoparticles using *Withania somnifera* leaf powder and silver nitrate. J. Photochem. Photobiol. B Biol..

[98] Bindhu M., Umadevi M. (2013). Synthesis of monodispersed silver nanoparticles using *Hibiscus cannabius* leaf extract and its antimicrobial activity. Spectrochim. Acta Part A Mol. Biomol. Spectrosc..

[99] Attar A., Yapaoz M.A. (2018). Biosynthesis of palladium nanoparticles using *Diospyros kaki* leaf extract and determination of antibacterial efficacy. Prep. Biochem. Biotechnol..

[100] Mavukkandy M.O., Chakraborty S., Abbasi T., Abbasi S.A. (2016). A Clean-Green Synthesis of Platinum Nanoparticles Utilizing a Pernicious Weed Lantana (*Lantana Camara*). Am. J. Eng. Appl. Sci..

[101] Narendhran S., Manikandan M., & Shakila P. (2019). Antibacterial, antioxidant properties of *Solanum trilobatum* and sodium hydroxide-mediated magnesium oxide nanoparticles: A green chemistry approach. Bull. Mater. Sci..

[102] Tahir K., Nazir S., Li B., Ahmad A., Nasir T., Khan A.U., Shah S.A.A., Khan Z.U.H., Yasin G., Hameed M.U. (2016). *Sapium sebiferum* leaf extract mediated synthesis of palladium nanoparticles and in vitro investigation of their bacterial and photocatalytic activities. J. Photochem. Photobiol. B Biol..

[103] Sathishkumar M., Sneha K., Yun Y.S. (2009). Palladium nanocrystal synthesis using *Curcuma longa* tuber extract. Int. J. Mater. Sci..

[104] Surendra T., Roopan S.M., Arasu M.V., Al-Dhabi N.A., Rayalu G.M. (2016). RSM optimized *Moringa oleifera* peel extract for green synthesis of *M. oleifera* capped palladium nanoparticles with antibacterial and hemolytic property. J. Photochem. Photobiol. B Biol..

[105] Basnet P., Chanu T.I., Samanta D., Chatterjee S. (2018). A review on bio-synthesized zinc oxide nanoparticles using plant extracts as reductants and stabilizing agents. J. Photochem. Photobiol. B Biol..

[106] Akbarian M., Mahjoub S., Elahi S.M., Zabihi E., Tashakkorian H., Elahi M. (2019). Appraisal of the biological aspect of Zinc oxide nanoparticles prepared using extract of *Camellia sinensis* L.. Mater. Res. Express.

[107] Azizi S., Mohamad R., Bahadoran A., Bayat S., Rahim R.A., Ariff A., Saad W.Z. (2016). Effect of annealing temperature on antimicrobial and structural properties of bio-synthesized zinc oxide nanoparticles using flower extract of *Anchusa italica*. J. Photochem. Photobiol. B Biol..

[108] Supraja N., Prasad T.N.V.K.V., Krishna T.G., David E. (2015). Synthesis, characterization, and evaluation of the antimicrobial efficacy of *Boswellia ovalifoliolata* stem bark-extract-mediated zinc oxide nanoparticles. Appl. Nanosci..

[109] Ringe E. (2020). Shapes, plasmonic properties, and reactivity of magnesium nanoparticles. J. Phys. Chem. C.

[110] Ramanujam K., Sundrarajan M. (2014). Antibacterial effects of biosynthesized MgO nanoparticles using ethanolic fruit extract of *Emblica officinalis*. J. Photochem. Photobiol. B Biol..

[111] Vanathi P., Rajiv P., Sivaraj R. (2016). Synthesis and characterization of *Eichhornia*-mediated copper oxide nanoparticles and assessing their antifungal activity against plant pathogens. Bull. Mater. Sci..

[112] Naika H.R., Lingaraju K., Manjunath K., Kumar D., Nagaraju G., Suresh D., Nagabhushana H. (2015). Green synthesis of CuO nanoparticles using *Gloriosa superba* L. extract and their antibacterial activity. J. Taibah Univ. Sci..

[113] Santhoshkumar T., Rahuman A.A., Jayaseelan C., Rajakumar G., Marimuthu S., Kirthi A.V., Velayutham K., Thomas J., Venkatesan J., Kim S.-K. (2014). Green synthesis of titanium dioxide nanoparticles using *Psidium guajava* extract and its antibacterial and antioxidant properties. Asian Pac. J. Trop. Med..

[114] Kirthi A.V., Jayaseelan C., Rahuman A. (2013). Biosynthesis and characterization of different nanoparticles and its larvicidal activity against human disease vectors. Mar. Biomater..

[115] Hu R., Beguiristain T., De Junet A., Leyval C. (2020). Bioavailability and transfer of elevated Sm concentration to alfalfa in spiked soils. Environ. Sci. Pollut. Res..

[116] Rezaee A. (2018). Accumulation and Toxicity of Lanthanum and Neodymium in Horticultural Plants. Ph.D. Thesis.

[117] Khan Z.U.H., Sadiq H.M., Shah N.S., Khan A.U., Muhammad N., Hassan S.U., Tahir K., Safi S.Z., Khan F.U., Imran M. (2019). Greener synthesis of zinc oxide nanoparticles using *Trianthema portulacastrum* extract and evaluation of its photocatalytic and biological applications. J. Photochem. Photobiol. B Biol..

[118] Ogunyemi S.O., Abdallah Y., Zhang M., Fouad H., Hong X., Ibrahim E., Masum M.I., Hossain A., Mo J., Li B. (2019). Green synthesis of zinc oxide nanoparticles using different plant extracts and their antibacterial activity against *Xanthomonas oryzae* pv. oryzae. Artif. Cells Nanomed. Biotechnol..

[119] Sukri S.N.A.M., Shameli K., Wong M.M.-T., Teow S.-Y., Chew J., Ismail N.A. (2019). Cytotoxicity and antibacterial activities of plant-mediated synthesized zinc oxide (ZnO) nanoparticles using *Punica granatum* (pomegranate) fruit peels extract. J. Mol. Struct..

[120] Nemati S., Hosseini H.A., Hashemzadeh A., Mohajeri M., Sabouri Z., Darroudi M., Oskuee R.K. (2019). Cytotoxicity and photocatalytic applications of biosynthesized ZnO nanoparticles by *Rheum turketanicum* rhizome extract. Mater. Res. Express.

[121] Sharmila G., Thirumarimurugan M., Muthukumaran C. (2018). Green synthesis of ZnO nanoparticles using *Tecoma castanifolia* leaf extract: Characterization and evaluation of its antioxidant, bactericidal and anticancer activities. Microchem. J..

[122] Hameed S., Khalil A.T., Ali M., Numan M., Khamlich S., Shinwari Z.K., Maaza M. (2019). Greener synthesis of ZnO and Ag–ZnO nanoparticles using *Silybum marianum* for diverse biomedical applications. Nanomedicine.

[123] Altikatoglu M., Attar A., Erci F., Cristache C.M., Isildak I. (2017). Green synthesis of copper oxide nanoparticles using *Ocimum basilicum* extract and their antibacterial activity. Fresenius Environ. Bull..

[124] Pagar K., Ghotekar S., Pagar T., Nikam A., Pansambal S., Oza R., Sanap D., Dabhane H. (2020). Antifungal activity of biosynthesized CuO nanoparticles using leaves extract of *Moringa oleifera* and their structural characterizations. Asian J. Nanosci. Mater..

[125] Ullah A.M., Tamanna A.N., Hossain A., Akter M., Kabir M.F., Tareq A.R., Kibria A.F., Kurasaki M., Rahman M.M., Khan M.N. (2019). In vitro cytotoxicity and antibiotic application of green route surface modified ferromagnetic TiO_2_ nanoparticles. RSC Adv..

[126] Rueda D., Arias V., Zhang Y., Cabot A., Agudelo A.C., Cadavid D. (2020). Low-cost tangerine peel waste mediated production of Titanium Dioxide Nanocrystals: Synthesis and characterization. Environ. Nanotechnol. Monit. Manag..

[127] Küünal S., Rauwel P., Rauwel E. (2018). Plant extract mediated synthesis of nanoparticles. Emerging Applications of Nanoparticles and Architecture Nanostructures.

[128] Parham S., Wicaksono D.H.B., Bagherbaigi S., Lee S.L., Nur H. (2016). Antimicrobial Treatment of Different Metal Oxide Nanoparticles: A Critical Review. J. Chin. Chem. Soc..

[129] Qamar S.U.R., Ahmad J.N. (2021). Nanoparticles: Mechanism of biosynthesis using plant extracts, bacteria, fungi, and their applications. J. Mol. Liq..

[130] Saka R., Chella N. (2020). Nanotechnology for delivery of natural therapeutic substances: A review. Environ. Chem. Lett..

[131] Sharma V., Kaushik S., Pandit P., Dhull D., Yadav J.P., Kaushik S. (2018). Green synthesis of silver nanoparticles from medicinal plants and evaluation of their antiviral potential against chikungunya virus. Appl. Microbiol. Biotechnol..

[132] Singh A., Gautam P.K., Verma A., Singh V., Shivapriya P.M., Shivalkar S., Sahoo A.K., Samanta S.K. (2020). Green synthesis of metallic nanoparticles as effective alternatives to treat antibiotics resistant bacterial infections: A review. Biotechnol. Rep..

[133] Singh P., Kim Y.-J., Zhang D., Yang D.-C. (2016). Biological Synthesis of Nanoparticles from Plants and Microorganisms. Trends Biotechnol..

[134] Sunny N.E., Kaviya A., Kumar S.V. (2022). Mechanistic approach on the synthesis of metallic nanoparticles from microbes. Agri-Waste and Microbes for Production of Sustainable Nanomaterials.

[135] Nweze J.A., Mbaoji F.N., Huang G., Li Y., Yang L., Zhang Y., Huang S., Pan L., Yang D. (2020). Antibiotics Development and the Potentials of Marine-Derived Compounds to Stem the Tide of Multidrug-Resistant Pathogenic Bacteria, Fungi, and Protozoa. Mar. Drugs.

